# Gene Networks Underlying the Resistance of *Bifidobacterium longum* to Inflammatory Factors

**DOI:** 10.3389/fimmu.2020.595877

**Published:** 2020-11-16

**Authors:** Vladimir A. Veselovsky, Marina S. Dyachkova, Egor A. Menyaylo, Polina S. Polyaeva, Evgenii I. Olekhnovich, Egor A. Shitikov, Dmitry A. Bespiatykh, Tatiana A. Semashko, Artem S. Kasianov, Elena N. Ilina, Valeriy N. Danilenko, Ksenia M. Klimina

**Affiliations:** ^1^Department of Molecular Biology and Genetics, Federal Research and Clinical Center of Physical-Chemical Medicine of Federal Medical Biological Agency, Moscow, Russia; ^2^Department of Biotechnology, Vavilov Institute of General Genetics Russian Academy of Sciences, Moscow, Russia; ^3^Phystech School of Biological and Medical Physics, Moscow Institute of Physics and Technology (National Research University), Dolgoprudny, Russia; ^4^Laboratory of Plant Genomics, The Institute for Information Transmission Problems of the Russian Academy of Sciences (Kharkevich Institute), Moscow, Russia; ^5^Faculty of Ecology, International Institute for Strategic Development of Sectoral Economics Peoples’ Friendship University of Russia (RUDN University), Moscow, Russia

**Keywords:** *Bifidobacterium longum*, RNA sequencing, transcriptome, pro-inflammatory cytokines, inflammatory process, transcription start site

## Abstract

As permanent residents of the normal gut microbiota, bifidobacteria have evolved to adapt to the host’s immune response whose priority is to eliminate pathogenic agents. The mechanisms that ensure the survival of commensals during inflammation and maintain the stability of the core component of the normal gut microbiota in such conditions remain poorly understood. We propose a new *in vitro* approach to study the mechanisms of resistance to immune response factors based on high-throughput sequencing followed by transcriptome analysis. This approach allowed us to detect differentially expressed genes associated with inflammation. In this study, we demonstrated that the presence of the pro-inflammatory cytokines IL-6 and TNFα to the growth medium of the *B. longum subsp. longum* GT15 strain changes the latter’s growth rate insignificantly while affecting the expression of certain genes. We identified these genes and performed a COG and a KEGG pathway enrichment analysis. Using phylogenetic profiling we predicted the operons of genes whose expression was triggered by the cytokines TNFα and IL-6 *in vitro*. By mapping the transcription start points, we experimentally validated the predicted operons. Thus, in this study, we predicted the genes involved in a putative signaling pathway underlying the mechanisms of resistance to inflammatory factors in bifidobacteria. Since bifidobacteria are a major component of the human intestinal microbiota exhibiting pronounced anti-inflammatory properties, this study is of great practical and scientific relevance.

## Introduction

Bifidobacteria are a key component of the commensal gut microbiota conferring considerable health benefits and supporting the normal functioning of the host organism (1).

Bifidobacteria exert many immunomodulatory properties that became the subject of many *in vitro* and *in vivo* studies (2). As the predominant component of the commensal microbiota of infants (3), bifidobacteria contribute significantly to the formation of the human immune system in the early stages of postnatal development (4, 5). The key factor in this process, ostensibly, is the ability to communicate with the immune system, which results in the regulation of both anti-inflammatory and pro-inflammatory cytokines and other factors of the immune response (2, 6).

As permanent residents of the normal gut microbiota, bifidobacteria have evolved to adapt the host’s immune response whose priority is to eliminate pathogenic agents. The mechanisms that ensure the survival of commensals during inflammation and maintain the stability of the core component of the normal gut microbiota in such conditions remain poorly understood.

Today, researchers have published a few works dedicated to the study of these mechanisms in commensal microorganisms. In one study, the authors suggested that the modification of lipopolysaccharides is a putative mechanism of such stability (7). The modification of lipopolysaccharides by the putative membrane-associated phospholipid phosphatase (BT1854 in *B. thetaiotaomicron*) increased the resistance to antimicrobial peptides of representatives of *Bacteroidetes*. Among actinobacteria, only *C. aerofaciens* became more resistant to antimicrobial peptides (7). Thus, the resistance mechanisms in bifidobacteria have not been studied. Since orthologs of the gene encoding the phosphatase BT1854 were not found in *Bifidobacterium* genomes [data not shown], we suspected that other genes could be involved in an altogether different pathway, which accounts for the formation of resistance in bifidobacteria.

Microorganisms can sense signaling molecules of the host’s immune system and respond to them by changing their growth rate as well as other characteristics (8). This is corroborated by the fact that interleukin-1 (IL-1) (9), interleukin-2 (IL-2) (10), granulocyte-macrophage colony-stimulating factor (GM-CSF) (10), interleukin-6 (11), interferon-γ (IFNγ) (12), and tumor necrosis factor α (TNFα) (13) all affect bacterial growth. The pronounced response of microorganisms to cytokines and other signaling molecules of the immune system implies changes in the transcription of genes involved in this response. Whole transcriptome analysis is one way to identify these genes.

Microarray-based technology, once the main tool to carry out transcriptome analysis, was replaced later by next-generation RNA-sequencing (RNA-Seq) technology. Today, RNA-seq is best suited for analyzing the fluctuating cellular transcriptome. Bacterial RNA-seq studies have refined considerably our understanding of bacterial gene expression (14). Today, studies on bifidobacteria using this technology are lacking. For example, transcriptome studies were performed: to identify candidate genes involved in the response to oxygen treatment in *B. longum* BBMN68 (3%, v/v) (15); to identify the genes and operons that are actively transcribed in *B. breve* UCC2003 during logarithmic growth (16); to examine strain-speciﬁc responses to tetracycline in *B. animalis* subsp. *lactis* Bl-04 and HN019 (17); to understand the utilization and metabolism of xylo-oligosaccharide in *B. adolescentis* 15703 and to identify the key regulatory-related genes (18); to identify genes potentially involved in the response to linoleic acid exposure in *B. breve* DSM 20213 (19); to analyze the changes in the gene expression profile following the exposure of the industrial probiotic strains *B. longum* JDM301 to acid and JDM301AR (20) to unravel the physiological bases accounting for these differences by pinpointing the transcriptional responses of *B. longum* NCC2705 and D2957 to sublethal H2O2 exposure (21).

In the present study, we performed transcriptome analysis of the *B. longum* subsp. *longum* GT15 strain to investigate the mechanisms lying behind the response of bifidobacteria to signaling molecules of the immune system. *B. longum* represents one of the most prevalent species of the *Bifidobacterium* genus in the gut microbiota of both infants and adults (22, 23). The genome of *B. longum subsp. longum GT15* was sequenced and characterized by us earlier (24).

Previously we characterized the *fn3* gene in *Bifidobacterium* genomes (25). The gene encodes a protein containing motifs complementary to the cytokine binding region of the gp-130 receptor. The ligand family of this human cytokine receptor includes IL-6 and other cytokines belonging to the IL-6 group (26). In addition, we previously confirmed the ability of FNIII domains of this protein to specifically bind to the cytokine TNFα (27). Therefore, we opted for the pro-inflammatory cytokines IL-6 and TNFα to assess the influence of immune response factors on the expression of *B. longum subsp. longum* GT15 genes.

The lists of differentially expressed genes (DEGs) revealed by the transcriptome analysis can potentially help us identify the genes involved in the mechanisms of resistance of commensal microorganisms, namely bifidobacteria, to pro-inflammatory immune factors.

## Materials and Methods

### Bacterial Strain and Growth Condition

In this work, we used the *B. longum* subsp*. longum* GT15 strain whose genome was sequenced and submitted to GenBank and assigned the accession no. CP006741 earlier (28). The cultivation of *B. longum* subsp*. longum* GT15 was carried out in Lactobacillus MRS Broth culture medium (HiMedia, India) supplemented with 0.5% cysteine (HiMedia, India). *B. longum* subsp*. longum* GT15 was cultured at 37°C under anaerobic conditions (HiAnaerobic System - Mark III, AnaeroHiGas Pack 3.5L; HiMedia, India).

### Exposure to Pro-Inflammatory Cytokines

A water solution of lyophilized recombinant human cytokines IL-6 and TNFα (Thermo Fisher Scientific, USA) was added to the experimental samples to a final concentration of [100pg/ml], [1ng/ml] and [10ng/ml]. *B. longum* subsp*. longum* GT15 grown in MRS-cys supplemented with an equal amount of water without cytokines was used as the control (10, 11). This experiment was conducted at 37°C under anaerobic conditions. Using a SmartSpec Plus spectrophotometer (Bio Rad), we measured the OD600 starting from timepoint 0 and ending at 24 h. The experiment was carried out in three biological replicates.

### RNA Extraction and Sequencing and Annotation

*B. longum* subsp*. longum* GT15 cells were collected in the middle of the log-phase in both experimental and control conditions. The cells were treated with RNA Protect Bacteria Reagent (QIAGEN). Total RNA extraction and purification was performed using the RNeasy Mini Kit (QIAGEN). Removal of residual gDNA was performed using the TURBO DNA-free Kit (Invitrogen) and the RNase-Free DNase Set (QIAGEN). The concentration and quality of the extracted RNA were checked using the Quant-iT RiboGreen RNA Assay Kit (Thermo Fisher Scientific) and the Agilent RNA 6000 Pico Kit (Agilent Technologies), respectively.

Total RNA (2 µg) was used for library preparation. Ribosomal RNA was removed from the total RNA using the RiboZero rRNA Removal Kit (Bacteria) (Epicentre/Illumina, Madison, USA) and libraries were prepared using the NEBNext^®^ Ultra II Directional RNA Library Prep Kit (NEB), according to the manufacturer protocol. Subsequently, RNA cleanup was performed with the RNA Clean XP kit (Beckman Coulter, Brea, USA). The library underwent a final cleanup using the Agencourt AMPure XP system (Beckman Coulter, Brea, USA) after which the libraries’ size distribution and quality were assessed using a high sensitivity DNA chip (Agilent Technologies). Libraries were subsequently quantified by Quant-iT DNA Assay Kit, High Sensitivity (Thermo Fisher Scientific). Finally, equimolar quantities of all libraries (12 pM) were sequenced by a high throughput run on the Illumina HiSeq using 2×100 bp paired-end reads and a 5% Phix spike-in control. Before loading the cBot system, the libraries were incubated at 98°C for 2 min and then cooled on ice to improve the hybridization of GC-rich sequences. The dataset of RNA-Seq analysis was deposited to the NCBI under the project name PRJNA628664.

### Data Processing and Analysis

Data processing and analysis were performed as previously described (29). Quality control of raw reads was carried out with FASTQC v0.11.5. Adaptors were trimmed with the Trimmomatic v0.33 tool. The Kallisto v0.46.0 software was used for mapping of reads and estimation of transcript abundance. Differential expression analysis was performed using edgeR v3.26.8 package, integrated in the Degust v4.1.1 web-tool. Only genes with count per million (CPM) ≥ 1 were analyzed further. Genes were filtered based on false discovery rate cutoff (FDR) ≤ 0.05 and minimum expression fold change (FC) ≥ 1. The plots were generated using the ggplot2 package in R. DEGs were then subjected to enrichment analysis of COG functions and Kyoto Encyclopedia of Genes and Genomes (KEGG) pathways. Bedtools genomecov (30) was used for coverage calculation. Using the moving average, coverage distribution tables were obtained. Data visualization was performed using GNU/R (31).

### Phylogenetic Profiling

Phylogenetic profiling included the identification of ortholog groups for *Bifidobacterium* genomes, the construction of binary vectors that indicate their presence (1) or absence (0) across several genomes, the construction of a matrix of pairwise distances between vectors, and the grouping of phylogenetic profiles (PP). The construction of PP was performed using the program OrthoFinder v.2.3.7 (32). A matrix of pairwise distances between PP was obtained using values based on the Jaccard Similarity Metric. PPs were constructed based on certain criteria: the distance values in a matrix of pairwise distances be minimal, genes must locate nearby and on the same strain, and all genes in the group must be downregulated or upregulated. Custom Python v.3.7.5 scripts have been used for the analysis (https://github.com/LabGenMO/phylo-profiling/blob/master/Identification%20of%20connected%20genes.ipynb).

### Preparation of 5′-Enriched Libraries for Transcriptional Start Sites Identification and Sequencing

Libraries for transcription start site (TSS) identification were prepared as described earlier (33) with some modifications. To prepare 5′-enriched libraries, we enriched whole transcriptome RNA by depleting ribosomal RNA (rRNA) using standard protocol RiboMinus (Thermo Fisher Scientific). mRNA was fragmented with Zn^2+^, end-repaired with T4 polynucleotide kinase according to the manufacturer protocol (Thermo Fisher Scientific) and treated with Terminator exonuclease (Epicentre/Illumina, Madison, USA). This procedure enriched RNA with 5’-triphosphate protected fragments. Then, the RNA was cleaned up with KAPA Pure Beads in a ratio of 1.8 to 1 and treated with RNA 5’ Pyrophosphohydrolase (RppH) (NEB) to remove the pyrophosphate groups and cleaned up again. The strand-specific libraries for Illumina sequencing were constructed from the obtained RNA. RNA was ligated with ds-adapters with protruding inner ends containing a random hexameric sequence (5’ ds-adapter sequences: RNA oligo 5’-AGUUCUACAGUCCGACGAUC-3’ and 5’-NNNNNNGATCGTCGGA-3’; 3’ ds-adapter sequences: kinated oligo 5’-pTGGAATTCTCGGGTGCCAAGG-3’ and 5’-GAGAATTCCANNNNNN-3’) using T4 DNA ligase (Thermo Fisher Scientific). Then, cDNA was generated using 5’-GGCACCCGAGAATTCCA-3’ primer and Maxima H Minus Reverse Transcriptase (Thermo Fisher Scientific) according to the manufacturer protocol. cDNA was clean up with KAPA Pure Beads, amplified with RNA PCR Index Primers from TruSeq Small RNA kit and was cleaned up using KAPA Pure Beads. Libraries were quantified by Quant-iT DNA Assay Kit, High Sensitivity (Thermo Fisher Scientific). The quality of libraries was evaluated using Bioanalyzer 2100 with high sensitivity DNA chip (Agilent Technologies).

Finally, equimolar quantities of all libraries (12 pM) were sequenced by high-throughput sequencing on Illumina HiSeq using 2×100 bp paired-end reads and a 5% PhiX spike-in control.

### Identification of Transcriptional Start Sites

Quality control on raw reads was carried out with FASTQC v0.11.5. Adaptors were trimmed using Trimmomatic v0.33. Bowtie2 was used to align short reads (34). SAM files processing was performed by samtools (35). Identification of TSS and subsequent analysis were performed from sam files with BAC-Browser (36) with standard parameters.

## Results

### Pro-Inflammatory Cytokines Have Little Effect on Growth of B. Longum Subsp. Longum GT15 Strain

To test the effects of pro-inflammatory cytokines on the growth of the bacterial culture, *B. longum* subsp*. longum* GT15 was incubated with various concentrations of IL-6 and TNFα while OD600 values were measured at different timepoints. The OD600 measurements under experimental and control conditions formed the growth curves of *B. longum* subsp*. longum* GT15 (**Figure S1**). We showed that the presence of IL-6 [100pg/ml] and TNFα [10 ng/ml] in the growth medium led to moderate changes in the growth rate of the culture (**Figure S1**). We used those same cultures for the subsequent transcriptome analysis.

### Differences in Gene Transcription Profiles

**Figure 1:**
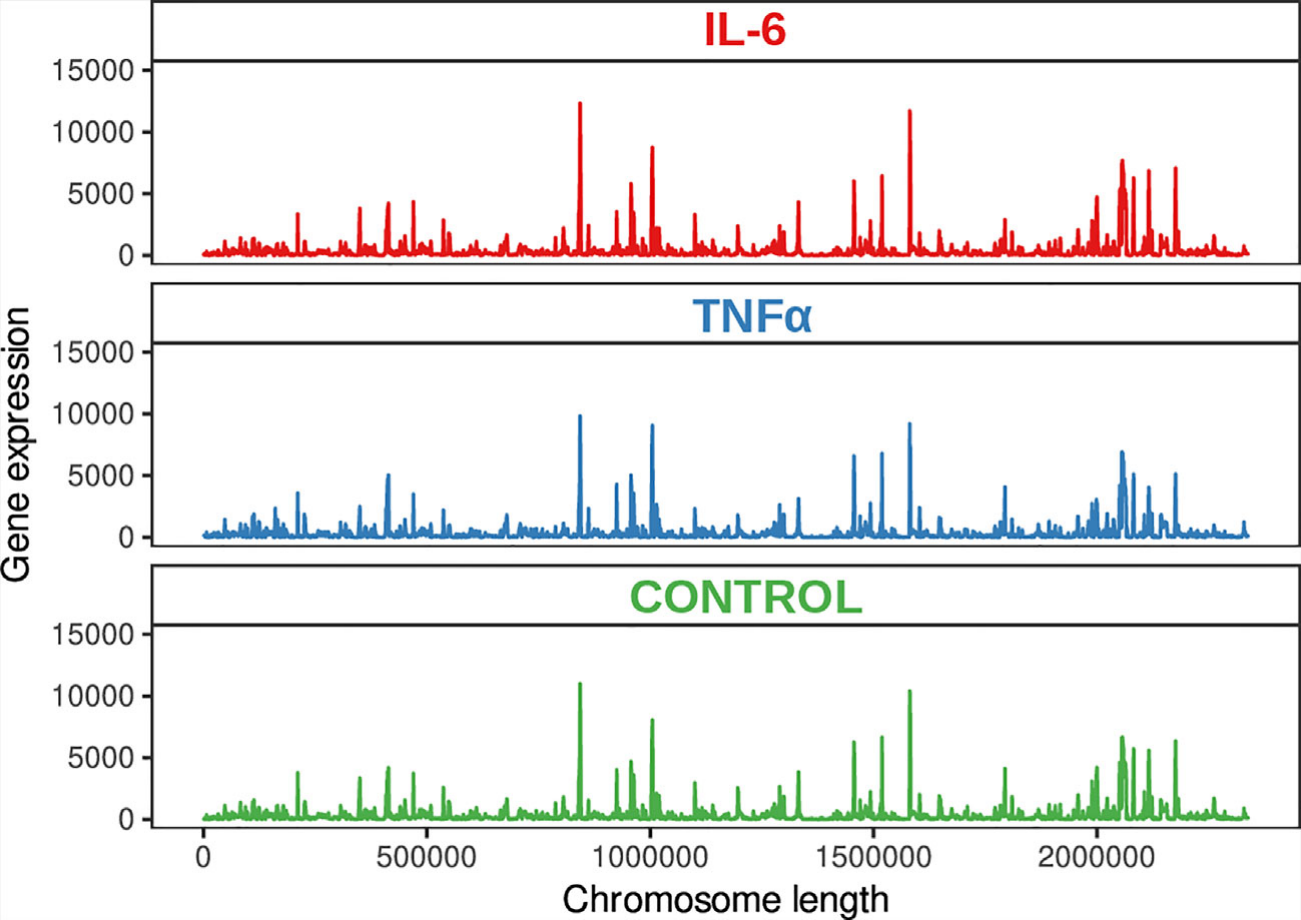
Genome visualization shows the transcriptional map of *B. longum subsp. longum* GT15.

To further understand the mechanism underlying the response of bifidobacteria to inflammation, we applied Illumina-sequencing technology for sequencing the whole transcriptome from three independent biological replicates of *B. longum* subsp*. longum* GT15 at the exponential growth phase (**Figure 1**).

**Figure 2:**
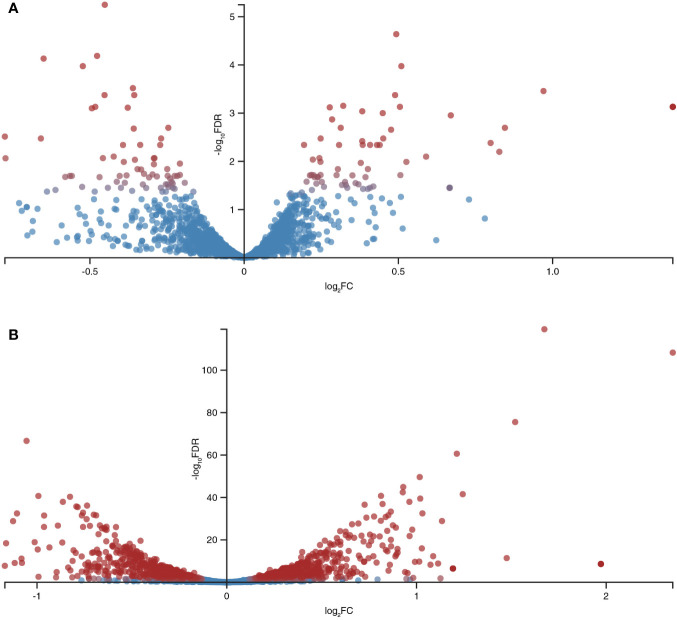
Global differentially expressed genes (DEGs) in *B. longum subsp. longum* GT15 resulting from **(A)** exposure to IL-6, **(B)** exposure to TNFα. Red dots: DEGs; blue dots: not DEGs.

A total of 12,602,595, 11,947,362, and 12,045,570 unique reads were obtained for the samples supplemented with the pro-inflammatory cytokines IL-6 [100pg/mL] and TNFα [10ng/mL] and a reference sample without pro-inflammatory cytokines (control), respectively. After filtering out poor quality reads, the number of effective reads mapped to the genome of *B. longum* subsp*. longum* GT15 was reduced to 11,283,525, 10,683,238, and 10,794,398, respectively. Genes that were significantly differentially expressed (based on a fold change of at least two [log 2 ratio <−1 or >1] and a t-test P-value < 0.001) in response to the presence of cytokines were singled out: a total number of 130 DEGs in the sample of *B. longum* subsp*. longum* GT15 grown with IL-6 compared to the control sample, including 68 downregulated and 62 upregulated genes and a total number of 1017 DEGs for the sample of *B. longum* subsp*. longum* GT15 grown with TNFα compared to the control sample, among which 505 genes were downregulated and 512 genes – upregulated (**Figure 2**, **Tables S1**).

The fold change (FC) in the expression of genes affected by the exposure of *B. longum* subsp*. longum* GT15 to IL-6 varied within the range −2>FC<2. In the case of exposure to TNFα, the FC in the expression of 992 genes fell within the range −2>FC<2 and the FC in expression of 25 genes was above 2. The levels of expression of these 25 genes (upregulated and downregulated) are presented in **Table 1**.

**Table 1:** Significantly upregulated and downregulated genes (FC≥ 2) upon exposure of *B. longum subsp. longum* GT15 to TNFα compared to controls by RNAseq.

Gene no	Annotation	Log_2_ (FC)
BLGT_RS00625	Hsp20/alpha crystalline family protein	5,10
BLGT_RS00855	Hypothetical protein	2,18
BLGT_RS01135	PEGA domain-containing protein	2,37
BLGT_RS01265	Uridylyltransferase	2,02
BLGT_RS01310	Sugar ABC transporter substrate-binding protein	2,20
BLGT_RS02915	ABC transporter substrate-binding protein	2,04
BLGT_RS02940	LacI family DNA-binding transcriptional regulator	2,32
BLGT_RS02975	Sugar ABC transporter substrate-binding protein	2,87
BLGT_RS03150	LamB/YcsF family protein	2,13
BLGT_RS03250	Zinc ABC transporter solute-binding protein	3,19
BLGT_RS03890	CarD family transcriptional regulator	−2,18
BLGT_RS04345	YccF domain-containing protein	−2,15
BLGT_RS04350	Hypothetical protein	−2,17
BLGT_RS04520	DUF4418 family protein	−2,24
BLGT_RS04525	ABC transporter permease	−2,12
BLGT_RS07100	SPFH domain-containing protein	2,02
BLGT_RS07650	DedA family protein	−2,02
BLGT_RS07990	AmmeMemoRadiSam system protein B	2,03
BLGT_RS08190	Sugar ABC transporter substrate-binding protein	2,10
BLGT_RS08205	ROK family glucokinase	−2,08
BLGT_RS08485	Sugar ABC transporter permease	2,04
BLGT_RS08615	Circularly permuted type 2 ATP-grasp protein	2,78
BLGT_RS08920	tRNA-Met	−2,11
BLGT_RS09910	Signal recognition particle sRNA small type	2,16
BLGT_RS09925	Hypothetical protein	−2,25

One of the most differentially expressed 25 genes was BLGT_RS08205, a ROK family glucokinase, encoding the NagC family transcriptional regulator. This gene is involved in many processes: carbohydrate metabolism (glycolysis/gluconeogenesis, galactose metabolism, starch and sucrose metabolism, amino sugar and nucleotide sugar metabolism); biosynthesis of other secondary metabolites (streptomycin biosynthesis, neomycin, kanamycin and gentamicin biosynthesis. Among the remaining genes, seven genes (BLGT_RS08190, BLGT_RS02975, BLGT_RS01310, BLGT_RS08485, BLGT_RS02915, BLGT_RS03250, BLGT_RS04525) encoded transporters involved in signaling and cellular processes, two genes (BLGT_RS03890, BLGT_RS00625) were involved in genetic information processing and the genes BLGT_RS03150, BLGT_RS08190, BLGT_RS01265, BLGT_RS08920 were involved in glutathione metabolism, membrane transport, signal transduction and translation, respectively.

### KEGG Pathway Mapping of DEGs

**Figure 3:**
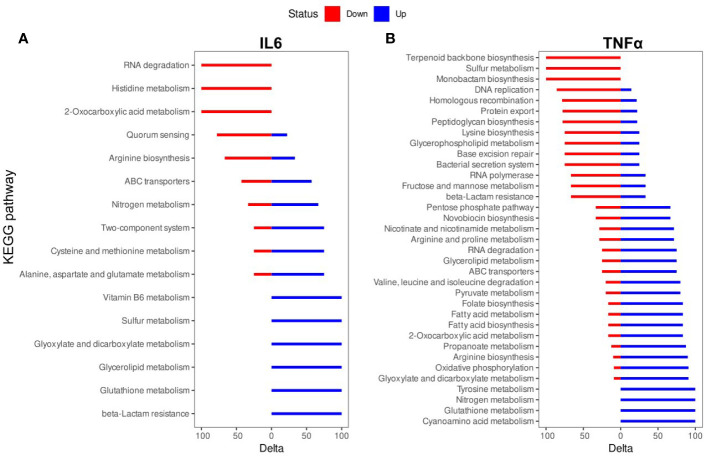
Kyoto Encyclopedia of Genes and Genomes (KEGG) pathway enrichment analysis of differentially expressed genes (DEGs): **(A)** IL-6 vs. control; **(B)**. TNFα vs. control. The names of KEGG pathways are placed along the Y-axis. The X- axis represents the values of the Delta. Downregulated genes are shown in red color. Upregulated genes are show in blue color.

The biologically significant DEGs were further analyzed by Kyoto Encyclopedia of Genes and Genomes (KEGG) pathways. As predicted by KEGG, 34 pathways were altered by IL-6 (**Figure 3A**, **Table S2**) and 87 pathways were altered by TNFα (**Figure 3B**, **Table S2**). A detailed representation of these pathways is given in **Figure 3**.

The TNFα-induced genes were involved in cyanoamino acid metabolism (3 (overexpressed genes)/4 (genes in the pathway)), glutathione metabolism (4/7), nitrogen metabolism (4/6), tyrosine metabolism (3/4), glyoxylate and dicarboxylate metabolism (10/14), oxidative phosphorylation (10/12), arginine biosynthesis (9/12), propanoate metabolism (7/12), 2-oxocarboxylic acid metabolism (10/21), fatty acid biosynthesis (5/7), fatty acid metabolism (5/7), folate biosynthesis (5/7), pyruvate metabolism (8/15), valine, leucine and isoleucine degradation (4/6), ABC transporters (21/54), glycerolipid metabolism (3/7), RNA degradation (6/10), arginine and proline metabolism (5/10), nicotinate and nicotinamide metabolism (5/13), novobiocin biosynthesis (2/4), pentose phosphate pathway (6/17). The TNFα-suppressed genes were involved in beta-Lactam resistance (4/7), fructose and mannose metabolism (2/4), RNA polymerase (2/4), bacterial secretion system (6/9), base excision repair (3/7), glycerophospholipid metabolism (3/6), lysine biosynthesis (6/11), peptidoglycan biosynthesis (7/17), protein export (7/12), DNA replication (6/13), homologous recombination (11/22), monobactam biosynthesis (3/4), sulfur metabolism (2/2), terpenoid backbone biosynthesis (5/9) (**Figure 3B**).

### Functional Annotation of DEGs

**Figure 4:**
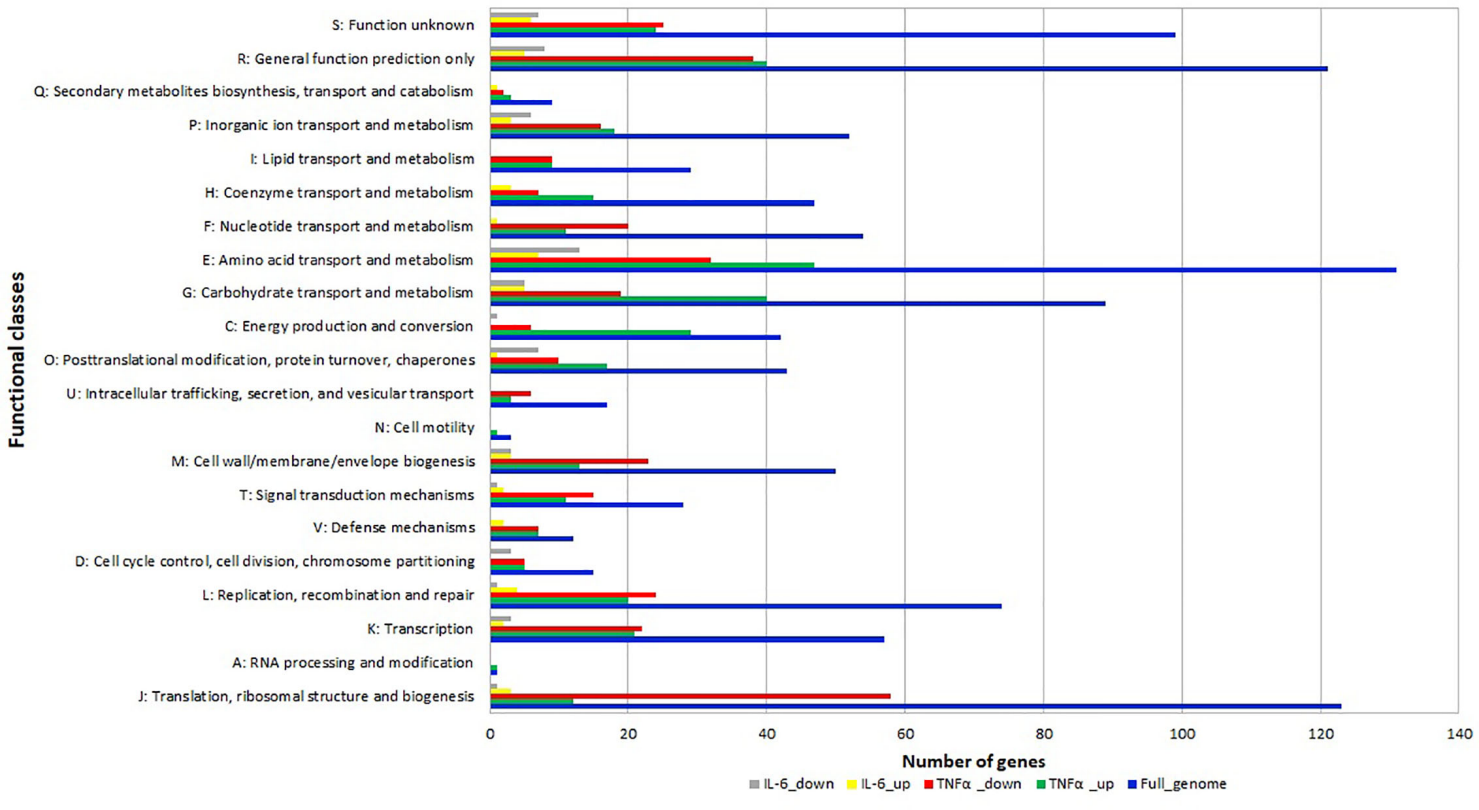
Relative abundance of transcripts grouped into Clusters of Orthologous Groups (COG) functional categories. Functional classification of genes with statistically significant increase (green bar for TNFα and yellow bar for IL-6) or decrease (red bar for TNFα and gray bar for IL-6) in mRNA level compared to controls.

The differentially expressed genes were grouped into functional categories according to the Clusters of Orthologous Groups (COG) classification system. COG categories H (coenzyme metabolism), E (amino acid transport and metabolism), G (carbohydrate transport and metabolism), C (energy production and conversion), O (posttranslational modification, protein turnover, chaperones) had a high number of upregulated genes after TNFα treatment compared to controls (**Figure 4**) whereas F (nucleotide transport and metabolism), U (intracellular trafficking, secretion, and vesicular transport), M (cell wall/membrane/envelope biogenesis), T (signal transduction mechanisms) L (replication, recombination and repair), K (transcription) J (translation, ribosomal structure and biogenesis) had a high number of downregulated genes after TNFα treatment compared to controls (**Figure 4**).

In addition, many genes in categories O (posttranslational modification, protein turnover, chaperones) and E (amino acid transport and metabolism) were also downregulated upon exposure to IL-6.

### Evolutionarily Stable Groups of Genes and Transcriptional Organization of DEGs

We used phylogenetic profiling to identify putative operons among the DEGs. We benefited from the open-source RefSeq database to analyze 130 genome sequences belonging to different *Bifidobacterium *species (**Table S3**). A phylogenetic profile (PP) is a binary vector describing the presence or absence of the coding sequence of a protein in a set of genomes of a group of organisms (37). It has been proposed that genes involved in the same biological pathway, especially genes in operons, are subjected to the same gain and loss evolutionary events. Therefore, genes with similar PPs are potential functional partners.

**Figure 5:**
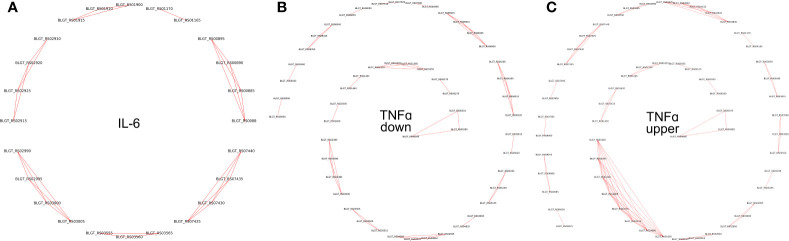
Operons of genes whose expression depends on the presence of **(A)** IL-6 and **(B)** TNFα downregulated **(C)** TNFα upregulated cytokines in the medium.

Pairs of genes having similar PPs (Jaccard distance less than 0.001), located on the same strand within 10,000 nucleotides, and having the same sign of differential (negative or positive) expression, were grouped together. Such groups were labeled as potential operons (**Table S4**, **Figure 5**).

To validate the predicted operons involved in the response to the pro-inflammatory cytokines IL-6 and TNFα in *B. longum* subsp*. longum* GT15, we carried out *de novo* identifications of transcriptional start sites (TSS). To carry out this task, we prepared RNA libraries enriched with 5’-fragments of mRNA and sequenced them using the high-throughput Illumina technology. The TSS were mapped with single-nucleotide resolution. The search for potential TSSs yielded total of 410 in *B. longum subsp. longum* GT15. Out of those, 38 TSSs belonged to operons of DEGs after exposure to TNFα, and 7 TSSs belonged to operons regulating DEGs resulting from exposure to IL-6 (**Table S3**, **Figure 5**).

Thus, we predicted and then experimentally validated the operons of genes whose expression was affected by the cytokines TNFα and IL-6 (**Figure 5**).

## Discussion

As the analysis of differentially expressed genes showed, TNFα altered the expression of a higher number of genes than IL-6. This may be due to the difference in the concentrations of used cytokines. According to earlier studies, the response of bacteria to treatment with cytokines may become dose-dependent after reaching a certain concentration (38). It is also possible that *B. longum* subsp*. longum* GT15 reacts differently to those two types of cytokines. Since cytokines are small peptide signaling molecules, the cytokine-mediated response of bacterial cells appears to be the result of interaction with receptor-like structures. Bacterial sensors enabling the recognition of immune signals have been described for pathogenic microorganisms (8). Moreover, we previously confirmed the ability of FNIII domains of this protein to specifically bind to cytokine TNFα but not IL-6 (27). Different cytokines, acting as ligands to specific receptors, could activate in bacterial cells different signaling pathways consisting of a different number of genes.

The results obtained in the *in vitro* experiment are subject to two different interpretations. One possible interpretation is that the response of *B. longum* subsp*. longum* GT15 to the pro-inflammatory cytokines is an adaptive reaction to stress aimed at maintaining normal functioning. Another interpretation is that the response of *B. longum* subsp*. longum* GT15 is intended to quench inflammation in the host organism, particularly in the gut. The cytokine-triggered release of mediators such as short-chain fatty acids, antioxidants, and other molecules that regulate the level of pro-inflammatory cytokines, supports well this hypothesis.

Nagasawa’s and Yu’s study highlighted the important role of hsp20 in reducing inflammation. The heat shock protein 20 (HSP20/HSPB6) belongs to the HSP family (HSPB) of small proteins with monomeric molecular masses ranging from 15 to 30 kDa. Overexpression of hsp20 leads to a marked decrease in TNF-α expression (39, 40). The transcription of the gene encoding *hsp20*, BLGT_RS00625, was upregulated 5-fold in the *B. longum* subsp*. longum* GT15 strain exposed to TNFα. Since the response of *B. longum* subsp*. longum* GT15 could be directed at bringing down TNFα levels, we suspect this mechanism to be one of the many ways used by bifidobacteria to dampen inflammation.

The transcription of ABC transporters (BLGT_RS02915, BLGT_RS02975, BLGT_RS03250, BLGT_RS04525, BLGT_RS08190, BLGT_RS08485) showed over two-fold upregulation. This can be explained by the cell’s struggle to maintain homeostasis during inflammation. An increase in the expression of ABC transporters, which facilitates the influx of sugars, leads to their accumulation in the cell followed by increased synthesis of ATP. In addition, a gene encoding Zinc ABC transporter (BLGT_RS03250) was 3,19-fold upregulated. Effective zinc absorption is crucial to an adequate response of the host’s immune system. One strategy adopted by the immune system is to decrease the concentration of zinc, iron and manganese (41–43). Therefore, TNF-α can stimulate the sequestration of zinc by bifidobacteria while it is still available.

The DedA family protein encoded by BLGT_RS07650 was also downregulated (**Table 1**). This protein has been shown to increase sensitivity of bifidobacteria to temperature and pH (44, 45). Hypersensitivity to temperatures can account for the increased expression of BLGT_RS00625.

Glutathione carries out many functions in biological systems among which are antioxidant activity, immune stimulation and cellular detoxification. Glutathione helps maintain the intracellular redox homeostasis, which ensures the protection of cells against oxidative damage. Most of the biological functions of glutathione depend on the conversion of reduced glutathione (GSH) to its oxidized form by the enzyme glutathione peroxidase and its transformation back to GSH by glutathione reductase. Glutathione, a potent reducing agent in the cell cytoplasm, is crucial for imparting antioxidant properties to bacteria. Glutathione defends cells against oxidative radicals, which pose a serious threat to the survival and performance of bacterial cells. Thus, both GSH synthesis and buildup in the cell could be correlated with the antioxidant potential of bacteria and their ability to grow in an aerobic environment, although other factors are also involved (46). The protective mechanism of GSH is suggested to be either its sacrificial action which prevents the rapid fall of intracellular pH or glutathionylation of the enzyme glyceraldehyde 3-phosphate dehydrogenase (GAPDH) which helps keeping the glycolysis rate under control. Epithelial cells require a continuous supply of GSH for proper functioning. It has been shown that exogenously supplied GSH not only maintains the necessary level in the intestine, but also provides protection against oxidizing agents such as tert-butyl hydroperoxide or menadione (47).

Ammonia has often been used as a marker of colonic bacterial protein metabolism. It is generally considered a potentially toxic metabolite causing direct damage to colonocytes. Nevertheless, ammonia is the main source of nitrogen for bacteria (48). Therefore, exposure to TNFα can signal to bifidobacteria an increase in the concentration of ammonia leading to increased expression of genes, involved in nitrogen metabolism. Similarly, the expression of genes involved in the metabolism of amino acids such as valine, leucine, arginine and proline could rise. The increased expression of genes involved in fatty acid metabolism could reflect the demand for acquiring resources for cell wall reparation during inflammation.

Recently, fecal metabolome analysis of 987 samples revealed altered pathways related to amino acid metabolism (e.g. aminoacyl-tRNA biosynthesis pathway, arginine biosynthesis pathway, and valine, leucine and isoleucine biosynthesis pathway), which correlated with changes in the core microbiome and inflammation (49, 50). Therefore, the changes in the expression of genes involved in biosynthesis and degradation of amino acids, in particular arginine, valine, leucine, and isoleucine, require a closer look in a future study.

Exposure to TNFα has also stimulated the expression of genes involved in propanoate metabolism. Propionate is potentially one of the main metabolites accounting for the probiotic action of bifidobacteria. Previously, propionate was shown to reduce inflammation by lowering blood pressure at the site of inflammation (51).

According to functional analysis, exposure to TNFα altered mainly the expression of genes included in energy pathways. This may be explained by the accumulation of resources caused by inflammation and activation of ATP-dependent ABC transporters to maintain homeostasis. Overall, IL-6 did not affect the expression of functional groups.

The observed effects of cytokines on *B. longum* subsp*. longum* GT15 were cumulative and prolonged, features that are conducive for the adaptation to the new conditions.

## Data Availability Statement

The datasets presented in this study can be found in online repositories. The names of the repository/repositories and accession number(s) can be found in the article/**Supplementary Material**.

## Author Contributions

VV, KK, and MD designed the experiments, performed the experiments, analyzed the samples, and contributed to the manuscript preparation. ES and DB analyzed the transcriptomic data. EM and AK analyzed the transcriptomic data and found COG. EO and KK contributed to KEGG pathway analysis and figures constructed. PP and MD made the phylogenetic profiling. VV and TS contributed to the transcriptional organization of DEGs. EI and VD contributed to the manuscript preparation. All authors contributed to the article and approved the submitted version.

## Funding

This research is supported by the Russian Science Foundation grant № 19-74-00146.

## Conflict of Interest

The authors declare that the research was conducted in the absence of any commercial or financial relationships that could be construed as a potential conflict of interest.

## Supplementary Material

Supplementary Figure 1 *B. longum subsp. longum* GT15 growth curves upon exposure to pro-inflammatory cytokines IL-6 and TNFα compared to cultivation without the cytokines.

Supplementary Table 1 The lists of DEGs.

Supplementary Table 2 KEGG pathway mapping of DEGs.

Supplementary Table 3 Species IDs.

Supplementary Table 4 Predicted operons.
