# Supplementary figures and images for: Gene Networks Underlying the Resistance of Bifidobacterium longum to Inflammatory Factors
Source: Front Immunol. 2020 Nov 16;11:595877. doi: 10.3389/fimmu.2020.595877 (PMC7701253; doi:10.3389/fimmu.2020.595877)

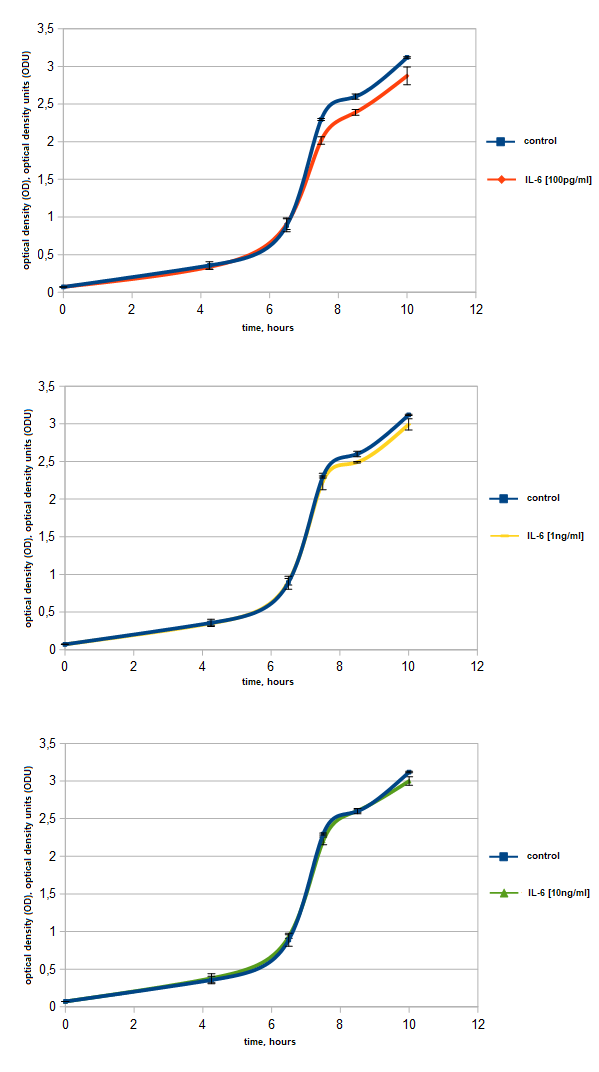

Supplement: Supplementary Figure 1 — B. longum subsp. longum GT15 growth curves upon exposure to pro-inflammatory cytokines IL-6 and TNFα compared to cultivation without the cytokines. [file DataSheet_1.zip › Supplementary/Figure_S1_IL-6_treatment.tiff]

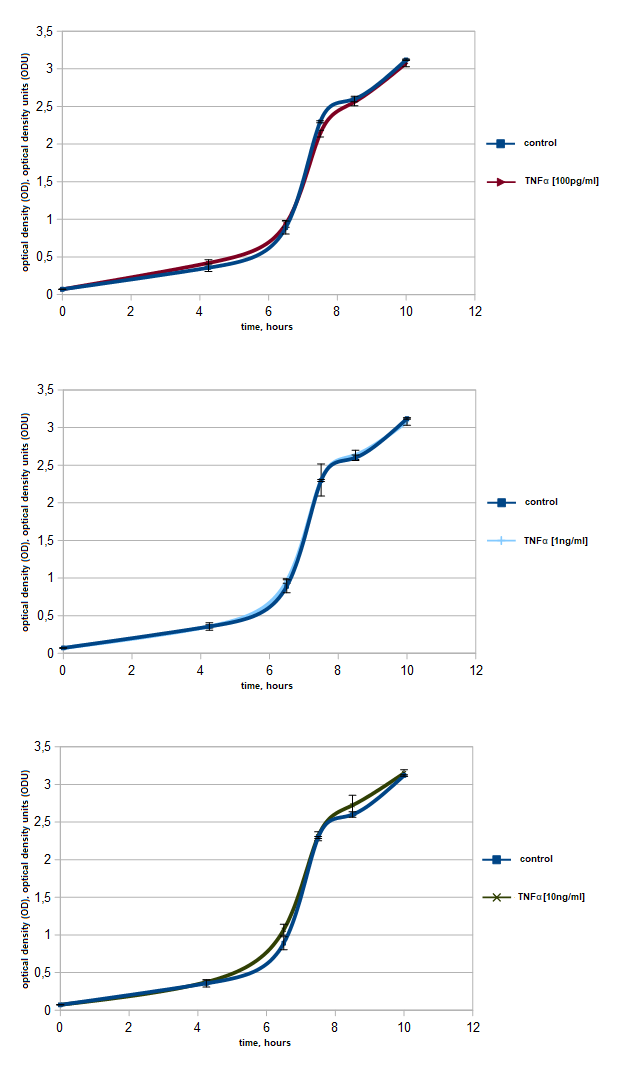

Supplement: Supplementary Figure 1 — B. longum subsp. longum GT15 growth curves upon exposure to pro-inflammatory cytokines IL-6 and TNFα compared to cultivation without the cytokines. [file DataSheet_1.zip › Supplementary/Figure_S1_TNF╬▒ _treatment.tiff]
